# Engaging with research impact assessment for an environmental science case study

**DOI:** 10.1038/s41467-019-12020-z

**Published:** 2019-10-04

**Authors:** Kirstie A. Fryirs, Gary J. Brierley, Thom Dixon

**Affiliations:** 1 Department of Environmental Sciences, Macquarie University, Sydney, NSW 2109 Australia; 2 School of Environment, University of Auckland, Auckland, 1010 New Zealand; 3 Research Services, Macquarie University, Sydney, NSW 2109 Australia

**Keywords:** Environmental impact, Research management

## Abstract

Impact assessment is embedded in many national and international research rating systems. Most applications use the Research Impact Pathway to track inputs, activities, outputs and outcomes of an invention or initiative to assess impact beyond scholarly contributions to an academic research field (i.e., benefits to environment, society, economy and culture). Existing approaches emphasise easy to attribute ‘hard’ impacts, and fail to include a range of ‘soft’ impacts that are less easy to attribute, yet are often a dominant part of the impact mix. Here, we develop an inclusive 3-part impact mapping approach. We demonstrate its application using an environmental initiative.

## Abstract (web summary)

Existing approaches to research impact assessment fail to include a range of soft impacts. The authors present a 3-part impact mapping approach and apply it to an environmental initiative. They highlight that support for realising research impact is vital, and call on researchers to be open to new ideas and avenues for creating impact from their work.

## Introduction

Universities around the World are increasingly required to demonstrate and measure the impact of their research beyond academia. The Times Higher Education (THE) World University Rankings now includes a measure of knowledge transfer and impact as an indicator of an institution’s quality and the THE World University Rankings released their inaugural University impact rankings in 2019. With the global rise of impact assessment, most nations adopt a variant of the Organisation for Economic Cooperation and Development (OECD) definition of impact^1^; “the contribution that research makes to the economy, society, environment or culture, beyond the contribution to academic research.” Yet research impact mapping provides benefits beyond just meeting the requirements for assessment^1^. It provides an opportunity for academics to reflect on and consider the impact their research can, and should, have on the environment, our social networks and wellbeing, our economic prosperity and our cultural identities. If considered at the development stage of research practices, the design and implementation of impact mapping procedures and frameworks can provide an opportunity to better plan for impact and create an environment where impact is more likely to be achieved.

**Fig. 1:**
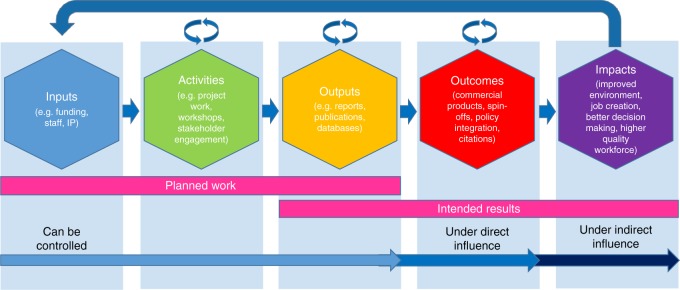
The Research Impact Pathway (modified from ref. ^2^)

Almost all impact assessments use variants of the Research Impact Pathway (Fig. 1) as the conceptual framework and model with which to document, measure and assess environmental, social, economic and cultural impacts of research^1^. This Pathway starts with inputs, followed by activities. Outputs and outcomes are produced and these lead to impact. Writing for *Nature Outlook: Assessing Science*, Morgan^2^ reported on how Australia’s Commonwealth Scientific and Research Organisation (CSIRO) mapped impact using this approach. However, the literature contains very few worked examples to guide academics and co-ordinators in the process of research impact mapping. This is particularly evident for environmental initiatives and innovations^3,4^.

Here we provide a new, 3-part impact mapping approach that can accommodate non-linearity in the impact pathway and can more broadly include and assess both ‘hard’ impacts, those that can be directly attributed to an initiative or invention, and ‘soft’ impacts, those that can be indirectly attributed to an initiative or invention. We then present a worked example for an environmental innovation called the River Styles Framework, developed at Macquarie University, Sydney, Australia. The River Styles Framework is an approach to analysis, interpretation and application of geomorphic insights into river landscapes as a tool to support management applications^5,6^. We document and map how this Framework has shaped, and continues to shape, river management practice in various parts of the world. Through mapping impact we demonstrate how the River Styles Framework has contributed to environmental, social and economic benefits at local, national and international scales. Cvitanovic and Hobday (2018)^3^ in Nature Communications might consider this case study a ‘bright spot’ that sits at the environmental science-policy-practice interface and is representative of examples that are seldom documented.

This case study is presented from the perspective of the researchers who developed the River Styles Framework, and the University Impact co-ordinator who has worked with the researchers to document and measure the impact as part of ex post assessment^1,7^. We highlight challenges in planning for impact, as the research impact pathway evolves and entails significant lag times^8^. We discuss challenges that remain in the mapping process, particularly when trying to measure and attribute ‘soft’ impacts such as a change in practice or philosophy, an improvement in environmental condition, or a reduction in community conflict to a particular initiative or innovation^9^. We then provide a personal perspective of the challenges faced and lessons learnt in applying and mapping research impact so that others, particularly in the environmental sciences and related interdisciplinary fields, can undertake similar exercises for their own research impact assessments.

## Brief background on research impact assessment and reporting

Historical reviews of research policy record long-term shifts towards incorporation of concerns for research impact within national funding agencies. In the 1970s the focus was on ‘research utilisation’^10^, more recently it has been on ‘knowledge mobilisation’^11^. The focus is always on seeking to understand the actual manner and pathways through which research becomes incorporated into policy, and through which research has an economic, social, cultural and environmental impact. Often these are far from linear circumstances, entailing multiple pathways.

Since the 1980s, higher education systems around the world have been transitioning to performance-based research funding systems (PRFS). The initial application of the PRFS in university contexts occurred as part of the first Research Assessment Exercise (RAE) in the United Kingdom in 1986^12^. PRFS systems have been designed to reward and perpetuate the highest quality research, presenting notionally rational criteria with which to support more intellectually competitive institutions^13^. The United Kingdom’s (UK) RAE was replicated in Australia as the Research Quality Framework (RQF), and more recently as the Excellence in Research for Australia (ERA) assessment. In 2010, 15 countries engaged in some form of PRFS^14^. These frameworks focus almost solely on academic research performance and productivity, rather than the contribution and impact that research makes to the economy, society, environment or culture.

In the last decade, research policy frameworks have increasingly focused on facilitating national prosperity through the transfer, translation and commercialisation of knowledge^15,16^, combined with the integration of research findings into government policy-making^17^. In 2009, the Higher Education Funding Council for England conducted a year-long review and consultation process regarding the structure of the Research Excellence Framework (REF)^18^. Following this review, in 2010 the Higher Education Funding Council for England (HEFCE) commissioned a series of impact pilot studies designed to produce narrative-style case studies by 29 higher education institutions. The pilot studies featured five units of assessment: clinical medicine, physics, earth systems and environmental sciences, social work and social policy, and English language and literature^12^. These pilot studies became the basis of the REF conducted in the UK in 2014^9,19^ with research impact reporting comprising a 20% component of the overall assessment.

In Canada, in 2009 and from 2014 the Canadian Academy of Health Sciences and Manitoba Research, respectively, developed an impact framework and narrative outputs to evaluate the returns on investment in health research^20,21^. Similarly the UK National Institute for Health Research (NIHR) regularly produces impact synthesis case studies^22^. In Ireland, in 2012, the Science Foundation Ireland placed research impact assessment at the core of its scientific and engineering research vision, called Agenda 2020^23^. In the United States, in 2016, the National Science Foundation, National Institute of Health, US Department of Agriculture, and US Environmental Protection Authority developed a repository of data and tools for assessing the impact of federal research and development investments^24^. In 2016–2017, the European Union (EU) established a high-level group to advise on how to maximise the impact of the EU’s investment in research and innovation, focussing on the future of funding allocation and the implementation of the remaining years of Horizon 2020^25^. In New Zealand, in 2017, the Ministry of Business, Innovation and Employment released a discussion paper proposing the introduction of an impact ‘pillar’ into the science investment system^26^. In 2020, Hong Kong will include impact assessment in their Research Assessment Exercise (RAE) for the first time^27^. Other countries including Denmark, Finland and Israel have scoped the use of research impact assessments of their major research programs as part of the Small Advanced Economies Initiative^28^.

In 2017, the Australian Research Council (ARC) conducted an Engagement and Impact Assessment Pilot (EIAP)^7^. While engagement is not analogous to impact, it is an evidential mechanism that elucidates the potential beneficiaries, stakeholders, and partners of academic research^12,16^. In addition to piloting narrative-style impact case study reporting, the EIAP characterised and mapped patterns of academic engagement with end users that create and enable research impact. The 2017 EIAP assessed a selection of disciplines for engagement, and a selection of disciplines for impact. Environmental science was a discipline selected for the impact pilot. These pilots became the basis for the Australian Engagement and Impact (EI) assessment in 2018^7^ that ran in parallel with the ERA, and from which the case study in this paper is drawn.

Research impact assessment does not just include ex post reporting that can feed into a national PRFS. A large component of academic impact assessment involves ex ante impact reporting in research funding applications. In both the UK and Australia, the perceived merit of a research funding application has been linked in part to its planning and potential for external research impact. In the UK this is labelled a ‘Pathways to Impact’ statement (used by the Research Council UK), in Australia this is an Impact statement (used by the ARC), with a national interest statement also implemented in 2018. These statements explicitly draw from the ‘pathway to impact’ model which simplifies a direct and linear relationship between research excellence, research engagement, and research impact^29^. These ex ante impact statements can be difficult for academics, especially early career researchers, if they do not understand the process, nature and timing of impact. This issue exists in ex post impact reporting and assessment as well, with many researchers finding it difficult to supply evidence that directly or indirectly links their research to impacts that may have taken decades to manifest^1,7,8^. Also, the simplified linearity of the Research Impact Pathway model makes it difficult to adequately represent the transformation of research into impact.

For research impact statements and assessments to be successful, researchers need to understand the patterns and pathways by which impact occurs prior to articulating how their own research project might achieve impact ex ante, or has had impact ex post. The quality of research impact assessment will improve if researchers and funding agencies understand the types and qualities of impact that can reasonably be expected to arise from a research project or initiative.

Given the plethora of interest in, and a growing global movement towards, both ex ante and ex post research impact assessment and reporting, it is surprising that very few published examples demonstrate how to map research impact. Even in the business, economics and corporate sectors where impact assessment and reporting is common practice^30–32^, very few published examples exist. This hinders prospects for researchers and co-ordinators to develop a more critical understanding of impact, inhibiting more nuanced understandings of the pathways to impact model. Mapping impact networks and recording a cartography of impact for research projects and initiatives provides an appropriate basis to conduct such tasks. This paper provides a new method by which this can be achieved.

## The research impact pathway and impact mapping

Many impact assessment frameworks around the world have common characteristics, often structured around the Research Impact Pathway model (Fig. 1). This model can be identified in a series of 2009 and 2016 Organisation for Economic Cooperation and Development (OECD) reports that investigated the mechanisms of impact reporting^1,33^. The Research Impact Pathway is presented as a sequence of steps by which impact is realised. This pathway can be visualised for an innovation or initiative using an impact mapping approach. It starts with inputs that can include funding, staff, background intellectual property and support structures (e.g., administration, facilities). This is followed by activities or the ‘doing’ elements. This includes the work of discovery (i.e., research) and the translation—i.e., courses, workshops, conferences, and processes of community and stakeholder engagement.

Outputs are the results of inputs and activities. They includes publications, reports, databases, new intellectual property, patents and inventions, policy briefings, media, and new courses or teaching materials. Inputs, activities and outputs can be planned and somewhat controlled by the researcher, their collaborators and their organisations (universities). Outcomes then occur under direct influence of the researcher(s) with intended results. This may include commercial products and licences, job creation, new contracts, grants or programs, citations of work, new companies or spin-offs and new joint ventures and collaborations.

Impacts (sometimes called benefits) tend to occur via uptake and use of an innovation or initiative by independent parties under indirect (or no) influence from the original researcher(s). Impacts can be ‘hard’ or ‘soft’ and have intended and unintended consequences. They span four main areas outside of academia, including environmental, social, economic and cultural spaces. Impacts can include improvements in environmental health, quality of life, changes in industry or agency philosophy and practice, implementation or improvement in policy, improvements in monitoring and reporting, cost-savings to the economy or industry, generation of a higher quality workforce, job creation, improvements in community knowledge, better inter-personal relationships and collaborations, beneficial transfer and use of knowledge, technologies, methods or resources, and risk-reduction in decision making.

## The challenge: applying the research impact pathway to map impact for a case study

The River Styles Framework^5,34^ aligns with UN Sustainable Development Goals of Life on Land and Clean Water and Sanitation that have a 2020 target to “ensure the conservation, restoration and sustainable use of terrestrial and inland freshwater ecosystems and their services” and a 2030 target to urgently “implement integrated water resources management at all levels”^35^.

The River Styles Framework is a catchment-scale approach to analysis and interpretation of river geomorphology^36^. It is an open-ended, generic approach for use in any landscape or environmental setting. The Framework has four stages (see refs. ^5,37–39^); (1) Analysis of river types, behaviour and controls, (2) Assessment of river condition, (3) Forecasting of river recovery potential, and (4) Vision setting and prioritisation for decision making.

River Styles Framework development, uptake, extension and training courses have contributed to a global change in river management philosophy and practice, resulting in improved on-ground river condition, use of geomorphology in river management, and end-user professional development. Using the River Styles Framework has changed the way river management decisions are made and the level of intervention and resources required to reach environmental health targets. This has been achieved through the generation of catchment-scale and regional-level templates derived from use of the Framework^6^. These templates are integrated with other biophysical science tools and datasets to enhance planning, monitoring and forecasting of freshwater resources^6^. The Framework is based on foundation research on the form and function of streams and their interaction with the landscape through which they flow (fluvial geomorphology)^5,40^.

The Framework has a pioneering structure and coherence due to its open-ended and generic approach to river analysis and interpretation. Going well beyond off-the-shelf imported manuals for river management, the Framework has been adopted because of its innovative approach to geomorphic analysis of rivers. The Framework is tailored for the landscape and institutional context of any given place to produce scaffolded, coherent and consistent datasets for catchment-specific decision making. Through on-ground communication of place-based results, the application of the Framework spans local, state, national and international networks and initiatives. The quality of the underlying science has been key to generating the confidence required in industry and government to adopt geomorphology as a core scientific tool to support river management in a range of geographical, societal and scientific contexts^6^.

The impact of this case study spans conceptual use, instrumental use and capacity building^4^ defined as ways of thinking and alerting policy makers and practitioners to an issue. Impact also includes direct use of research in policy and planning decisions, and education, training and development of end-users, respectively^4,41,42^. The River Styles Framework has led to establishment of new decision-making processes while also changing philosophy and practice so on-ground impacts can be realised.

Impact does not just occur at one point in time. Rather, it comes and goes or builds and is sustained. How this is represented and measured, particularly for an environmental case study, and especially for an initiative built around a Framework where a traditional ‘product’, ‘widget’, or ‘invention’ is not produced is challenging^4^. More traditional metrics-based indicators such as the number of lives saved or the amount of money generated cannot be deployed for these types of case studies^4,9^. It is particularly challenging to unravel the commercial value and benefits of adopting and using an initiative (or Framework) that is part of a much bigger, international paradigm shift in river management philosophy and practice.

Similarly, how do you measure environmental, social, economic or cultural impacts of an initiative where the benefits can take many years (and in the case of rivers, decades) to emerge, and how do you then link and attribute those impacts directly with the design, development, use and extension of that initiative in many different places at many different times? For the River Styles Framework, on-ground impacts in terms of improved river condition and recovery are occurring^43^, but other environmental, social and economic benefits may be years or decades away. Impactful initiatives in themselves often reshape the contextual setting that then frames the next phase of science and management practices which leads to further implications for policy and institutional settings, and for societal (socio-cultural) and environmental benefits. This is currently the case in assessing the impact of the River Styles Framework.

## The method: a new, 3-part impact mapping approach

**Fig. 2:**
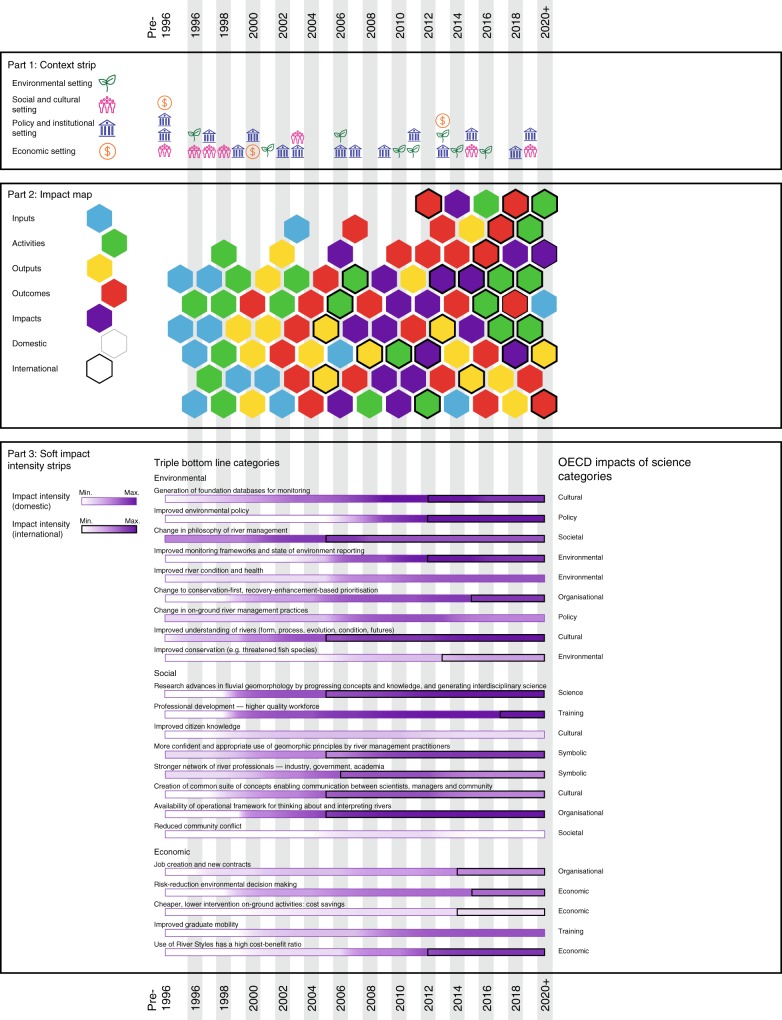
The research impact map for the River Styles Framework case study. This map contains 3 parts, a context strip, impact map and soft impact intensity strips

Using the River Styles framework as an environmental case study, Fig. 2 presents a 3-part impact mapping approach that contains (1) a context strip, (2) an impact map, and (3) soft impact intensity strips to capture the scope of the impact and the conditions under which it has been realised. This approach provides a template that can be used or replicated by others in their own impact mapping exercises^44^.

The cartographic approach to mapping impact shown in Fig. 2 provides a mechanism to display a large amount of complex information and interactions in a style that conveys and communicates an immediate snapshot of the research impact pathway, its components and associated impacts. The map can be analysed to identify patterns and interactions between components as part of ex post assessment, and as a basis for ex ante impact forecasting.

The 3-part impact map output is produced in an interactive online environment, acknowledging that impact maps are live, open-ended documents that evolve as new impacts emerge and inputs, activities, outputs and outcomes continue. The map changes when activities, outputs or outcomes that the developers had forgotten, or considered to be peripheral, later re-appear as having been influential to a stakeholder, community or network not originally considered as an end-user. Such activities, outputs and outcomes can be inserted into a live map to broaden its base and understand the impact. Also, by clicking on each icon on the map, pop-up bubbles contain details that are specific to each component of the case study. This functionality can also be used to journal or archive important information and evidence in the ‘back-end’ of the map. Such evidence is often required, or called upon, in research impact assessments. Figure 2 only provides a static reproduction of the map output for the River Styles Framework. The fully worked, interactive, River Styles Framework impact map can be viewed at https://indd.adobe.com/view/c9e2a270–4396–4fe3-afcb-be6dd9da7a36.

Context is a key driver of research impact^1,45^. Context can provide goals for research agendas and impact that feeds into ex ante assessments, or provide a lens through which to analyse the conditions within which certain impacts emerged and occurred as part of ex post assessment. Part 1 of our mapping approach produces a *context strip* that situates the case study (Fig. 2). This strip is used to document settings occurring outside of academia before, during and throughout the case study. Context can be local, national or global and examples can be gathered from a range of sources such as reports, the media and personal experience. For the River Styles case study only key context moments are shown. Context for this case study is the constantly changing communities of practice in global river restoration that are driven by (or inhibited by) the environmental setting (coded with a leaf symbol), policy and institutional settings (coded with a building symbol), social and cultural settings (coded with a crowd symbol), and economic settings (coded with a dollar symbol). For most case studies, these extrinsic setting categories will be similar, but others can be added to this part of the map if needed.

Part 2 of our mapping approach produces an impact map using the Research Impact Pathway (Fig. 1). This *impact map* (Fig. 2) documents the time-series of inputs (coded with a blue hexagon), activities (coded with a green hexagon), outputs (coded with a yellow hexagon), outcomes (coded with a red hexagon) and impacts (coded with a purple hexagon) that occurred for the case study. Heavier bordered hexagons and intensity strips represent international aspects and uptake. To start, only the primary inputs, activities, outputs and outcomes are mapped. A hexagon appears when there is evidence that an input, activity, output or outcome has occurred. Evidence includes event advertisements, reports, publications, website mentions, funding applications, awards, personnel appointments and communications products.

However, in conducting this standard mapping exercise it soon became evident that it is difficult to map and attribute impacts, particularly for an initiative that has a wide range of both direct and indirect impacts. To address this, our approach distinguishes between ‘hard’ impacts and ‘soft’ impacts. Hard impacts can be directly attributed to an initiative or invention, whereas soft impacts can be indirectly attributed to an initiative or invention. The inclusion of soft impacts is critical as they are often an important and sometimes dominant part of the impact mix. Both quantitative and qualitative measures and evidence can be used to attribute hard or soft impacts. There is not a direct one-to-one relationship between quantitative measurement of hard impacts and qualitative appraisal of soft impacts.

Hard impacts are represented as purple hexagons in the body of the impact map. For the River Styles Framework we have only placed a purple hexagon on the impact map where the impact can be ‘named’ and for which there is ‘hard’ evidence (in the form of a report, policy, strategic plan or citation) that directly mentions and therefore attributes the impact to River Styles. Most of these are multi-year impacts and the position of the hexagons on the map is noted at the first mention.

For many case studies, particularly those that impact on the environment, society and culture, attributing impact directly to an initiative or invention is not necessarily easy or straighforward. To address this our approach contains a third element, soft impact intensity strips (Fig. 2) to recognise, document, capture and map the extent and influence of impact created by an initiative or invention. This is represented as a heat intensity chart (coded as a purple bar of varying intenstiy) and organised under the environmental, social and economic categories that are often used to measure Triple-Bottom-Line (TBL) benefits in sustainability and research and development (R&D) reporting (e.g., refs. ^7,46^). Within these broad categories, soft impacts are categorised according to the dimensions of impacts of science used by the OECD^1^. These include environmental, societal, cultural, economic, policy, organisational, scientific, symbolic and training impacts. Each impact strip for soft impacts uses different levels of purple shading (to match the purple hexagon colour in the impact map) to visualise the timing and intensity of soft impacts. For the River Styles Framework, the intensity of the purple colour is used to show those impacts that have been most impactful (darker purple), the timing of initiation, growth or step-change in intensity of each impact, the rise and wane of some impacts and the longevity of others. A heavy black border is used to note the timing of internationalisation of some impacts. This heat intensity chart was constructed by quantitatively representing qualitative sentiment in testimonials, interviews, course evaluations and feedback, surveys and questionnaires, acknowledgements and recognitions, documentation of collaborations and networks, use of River Styles concepts, and reports on the development of spin-off frameworks. Quantitative representations of qualitative sentiment was achieved through using the methods of time-series keyword searches and expert judgement. These are just two methods by which the level of heat intensity can be measured and assigned^9^.

## The outcome: impact of the River Styles Framework case study

Figure 2, and its interactive online version, present the impact map for the River Styles Framework initiative and Table 1 documents the detail of the River Styles impact story from pre-1996 to post-2020. The distribution of colour-coded hexagons and the intensity of purple on the soft impact intensity strips on Fig. 2 demonstrates the development and maturation of the initiative and the emergence of the impact.

**Table 1:** The River Styles Framework impact story (pre-1996 to post-2020)

1996–2002	• Blue inputs, green activities and yellow outputs hexagons dominate • Intensive phase of gathering and establishing inputs including funding for foundational research, appointing researchers, and background intellectual property • Significant activity (green hexagons) framed around community and industry engagement, activities such as Town Hall meetings, on-ground applications, field days, and conference presentations
2002–2005	• Establishing the brand and setting quality control measures on use of the Framework • Intensive phase of output production (yellow hexagons) including publication of a book and e-book^5,49^, and launch of the River Styles website^34^ • Red outcome hexagons appear and intensify with development and delivery of professional short courses for industry and trademarking the River Styles brand • On-going foundational research on the Framework (green activity hexagons) via large research grants and appointment of post-doctoral fellows
2006–2007	• Purple impact hexagons appear for the first time in 2006, representing hard impact outside of academia. The first appears as River Styles is used in NSW Catchment Action Plans and in Tasmanian River Health Strategies. These plans are decadal in timeframe and several are renewed to 2024, acknowledging the longevity of River Styles impact in shaping river management practice and philosophy • Soft impacts emerge more intensely • International recognition of the Framework (heavier bordered hexagons) starts to emerge. Initially this occurs through the take-up and citation of research publications (yellow outputs), the running of new short courses and the conduct of international conference presentations (green activities) • In 2007 the River Styles Framework won the Macquarie University award for Research Innovation • In 2007 Land and Water Australia (LWA) undertook the first of two cost:benefit analyses, valuing the Framework at $35million. These outcomes (red hexagons) significantly bolstered the River Styles reputation
2008–2013	• Domestic consolidation of yellow outputs, red outcomes and purple impacts, and the start of both directed and independent international uptake • The number of purple impact hexagons increases during 2008–2015 and soft impacts intensify further • Domestically, impact is focussed on use of the Framework for monitoring, evaluation, prioritisation, decision-making and improving on-ground practices as part of National and State programs, strategic plans and policy. Some of this is led by changes to the New South Wales Water Act 2007 into which the Framework has been integrated as one assessment tool in water sharing plans, the Australian National Aquatic Ecosystem Classification Framework, and the High Ecological Value Aquatic Ecosystems evaluation • Yellow outputs include the publication of two more books, one of which ‘River Futures’ brings to Australia a team of international collaborators as part of the Upper Hunter River Rehabilitation Initiative (UHRRI) to observe practice and write on the state of river management in their countries^50^ • Other yellow outputs are co-published with NSW industry practitioners to document the impact of using River Styles in river management in NSW^6^ • River Styles presentations in India (2008), China (2009), Singapore (2009), Malaysia (2009), Poland (2013), Czech Republic (2013)
2014–2015	• The River Styles Framework now provides the powerboard into which new initiatives, approaches and inventions are being plugged^51^ • Internationally, the Framework is used in a range of river management initiatives (some driven by policy, some not). Some of this uptake is under direct influence and some is independent • The River Styles Framework inspired research activities for the REstoring rivers FOR effective catchment Management (REFORM) program for implementation in the European Water Framework Directive (WFD) and becomes the foundation Framework from which other approaches to analysis of river character, condition, recovery and prioritisation assessment tools are developed (^36,52–59^). These spin-off frameworks adopt River Styles principles and have a distinctly River Styles flavour • In the USA, the Columbia Habitat Monitoring Program (CHaMP) adopts the Framework for fish habitat mapping and monitoring. This sparks a wave of innovation and collaboration in the development of semi-automated tools for the geomorphic analyses of rivers^60^ • River Styles workshops and presentations occur in Sweden (2014), Spain (2014), Austria (2014), United States (2014) • Red outcomes focus on delivery of six more professional short courses, the demand for which continues to escalate. The portfolio of professional development courses is expanded and delivered domestically for the first time in 2014 • Funding for the River Styles Framework is largely external to the university system as agencies and industry invest in and apply the Framework. The only direct input (blue hexagon) is a seed grant to build a River Styles App • In 2010 the Framework was valued at $40 million in the second LWA cost:benefit analysis • Foundation research, expert advice and stakeholder engagement continues (green hexagons), totalling some 600 h per year
2016–2019 (and post-2020)	• Phase of extension into international markets, collaborations and uses • Only new purple impact hexagons are added to the map during this period. Impacts that emerged most intensively post-2006 continue in the background • Green activity hexagons re-appear, much like the 1996–2002 phase, but in an international context • Professional short courses run in Brazil in 2017^61^ • Foundation science continues, particularly internationally with new and emerging collaborations • Yellow outputs and red outcomes continue to be produced • By 2017, over 6500 books have been sold world-wide, the 2005 book reaches 800 citations and total citations for River Styles publications reaches nearly 3500 • In 2018, the River Styles Framework is chosen for use by The Nature Conservancy to inform river management and conservation in Colombia • In 2019 the NSW River Styles database is released via a Creative Commons license for use by any agency involved in river management • River Styles Short Courses delivered in India and Philippines

In the first phase (pre-1996–2002), blue inputs, green activities and yellow output hexagons dominate. The next phase (2002–2005) was an intensive phase of output production (yellow hexagons). It is during this phase that red outcome hexagons appear and intensify. From 2006, purple impact hexagons appear for the first time, representing hard impact outside of academia. Soft impacts also start to emerge more intensely (Fig. 2). 2008–2015 represents a phase of domestic consolidation of yellow outputs, red outcomes and purple impacts, and the start of international uptake. Some of this impact is under direct influence and some is independent of the developers of the River Styles Framework (Fig. 1). The number of purple impact hexagons is more intense during the 2008–2015 period and soft impacts intensify further. 2016–2018 (and beyond) represents a phase of extension into international markets, collaborations and impact (heavier bordered hexagons and intensity strips; Fig. 2). The domestic impacts that emerged most intensively post-2006 continue in the background. Green activity hexagons re-appear during this period, much like the 1996–2002 phase, but in an international context. Foundational science (green activity hexagons) re-emerge, particularly internationally with new collaborations. At the same time, yellow outputs and red outcomes continue.

For the River Styles case study the challenge still remains one of how to adequately attribute, measure and provide evidence for soft impacts^4^ that include:

- a change in river management philosophy and practice
- an improvement in river health and conservation of threatened species
- the provision of an operational Framework that provides a common and consistent approach to analysis
- the value of knowledge generation and databases for monitoring river health and informing river management decision-making for years to come
- the integration into, and improvement in, river management policy
- a change in prioritisation that reduces risk in decision-making and cost savings on-the-ground
- professional development to produce a better trained, higher quality workforce and increased graduate employability
- the creation of stronger networks of river professionals and a common suite of concepts that enable communication
- more confident and appropriate use of geomorphic principles by river management practitioners
- an improvement in citizen knowledge and reduced community conflict in river management practice

## Lessons learnt by applying research impact mapping to a real case study

When applying the Research Impact Pathway and undertaking impact mapping for a case study it becomes obvious that generating and realising impact is not a linear process and it is never complete, and in many aspects it cannot be planned^8,9,29^. Rather, the pathway has many highways, secondary roads, intersections, some dead ends or cul-de-sacs and many unexpected detours of interest along the way.

Cycles of input, activity, outputs, outcomes and impact occur throughout the process. There are phases where greater emphasis is placed on inputs and activities, or phases of productivity that produce outputs and outcomes, and there are phases where the innovation or initiative gains momentum and produces a flurry of benefits and impacts. However, throughout the journey, inputs, activities, outputs and outcomes are always occurring, and the impact pathway never ends. Some impacts come and go while others are sustained.

The saying “being in the right place at the right time with the right people” has some truth. Impact can be probabilistically generated ex ante by the researcher(s) regularly placing themselves and their outputs in key locations or ‘rooms’ and in ‘moments’ where the chance of non-academic translation is high^47^. Context is also critical^45^. Economic, political, institutional, social and environmental conditions need to come together if an innovation or initiative is to ‘get off the ground’, gain traction and lead to impact (e.g., Fig. 2). Ongoing and sustained support is vital. An innovation funded 10 years ago may not receive funding today, or an innovation funded today may not lead to impact unless the right sets of circumstances and support are in place. This is, in part, a serendipitous process that involves the calculated creation of circumstances aligned to evoke the ‘black swan’ event of impact^48^. The ‘black swan’ effect, coined by Nassem Nicholas Taleb, is a metaphor for an unanticipated event that becomes reinterpreted through the benefit of hindsight, or alternatively, an event that exists ‘outside the model’. For example, black swans were presumed not to exist by Europeans until they were encountered in Australia and scientifically described in 1790. Such ‘black swan’ events are a useful device in ex post assessment for characterising those pivotal moments when a research program translates into research impact. While the exact nature of such events cannot be anticipated, by understanding the ways in which ‘black swan’ events take place in the context of research impact, researchers can manufacture scenarios that optimise their probability of provoking a ‘black swan’ event and therefore translating their research project into research impact, albeit in an unexpected way. One ‘black swan’ event for the River Styles Framework occurred between 1996–2002 (Table 1). Initial motivations for developing the Framework reflected inappropriate use of geomorphic principles derived elsewhere to address management concerns for distinctive river landscapes and ecosystems in Australia. Although initial applications and testing of the Framework were local (regional-scale), advice by senior-level personnel in the original funding agency, Land and Water Australia (blue input hexagon in 1997; Fig. 2), suggested we make principles generic such that the Framework can be used in any landscape setting. The impact of this ‘moment’ was only apparent much later on, when the Framework was adopted to inform place-based, catchment-specific river management applications in various parts of the world.

What is often not recognised is the time lag in the research impact process^9^. Depending on the innovation or initiative, this is, at best, a decadal process. Of critical importance is setting the foundations for impact. The ‘gem of an idea’ needs to be translated into a sound program of research, testing (proof of concept), peer-review and demonstration. These foundations must generate a level of confidence in the innovation or initiative before uptake. A level of branding may be required to make the innovation or initiative stand out from the crowd. Drivers are required to incentivise academics, both internal and external to their University setting, encouraging them to go outside their comfort zone to apply and translate their research in ‘real-world’ settings. Maintaining passion, patience and persistence throughout the journey are some of the most hidden and unrecognised parts of this process.

Some impacts are not foreseeable and surprises are inevitable. Activities, outputs and outcomes that may initially have seemed like a dead end, often re-appear in a different context or in a different network. Other outputs or outcomes take off very quickly and are implemented with immediate impact. Catalytic moments are sometimes required for uptake and impact to be realised^8^. These surprises are particularly obvious when an innovation or initiative enters the independent uptake stage, called impact under indirect influence on Fig. 1. In this phase the originating researchers, developers or inventors are often absent or peripheral to the impact process. Other people or organisations have the confidence to use the innovation or initiative (as intended, or in some cases not as intended), and find new ways of taking the impact further. The innovation or initiative generates a life of its own in a snowball effect. Independent uptake is not easily measured, but it is a critical indicator of impact. Unless the foundations are solid and sound, prospects for sustained impact are diminished.

The maturity and type of impact also vary in different places at different times. This is particularly the case for innovations and initiatives where local and domestic uptake is strong, but international impact lags. Some places may be well advanced on the uptake part of the impact journey, firmly embedding the benefits while developing new extensions, add-ons and spin-offs with inputs and activities. Elsewhere, the uptake will only have just begun, such that outputs and outcomes are the primary focus for now, with the aim of generating impact soon. In some instances, authorities and practitioners are either unaware or are yet to be convinced that the innovation or initiative is relevant and useful for their circumstances. In these places the focus is on the inputs and activity phases necessary to generating outputs and outcomes relevant to their situation and context. Managing this variability while maintaining momentum is critical to creating impact.

## Future directions for the practice of impact mapping and assessment

The process of engaging with impact and undertaking impact mapping for an environmental case study has been a reflective, positive but challenging experience. Our example is typical of many of the issues that must be addressed when undertaking research impact mapping and assessments where both ‘hard’ and ‘soft’ impacts are generated. Our 3-part impact mapping approach helps deal with these challenges and provides a mechanism to visualise and enhance communication of research impact to a broad range of scientists and policy practitioners from many fields, including industry and government agencies, as well as citizens who are interested in learning about the tangible and intangible benefits that arise from investing in research.

Such impact mapping work cannot be undertaken quickly^44,45^. Lateral thinking is required about what research impact really means, moving beyond the perception in academia that outputs and outcomes equals impact^4,9,12^. This is not the case. The research impact journey does not end at outcomes. The real measure of research impact is when an initiative gains a ‘life of its own’ and is independently picked-up and used for environmental, social or economic benefit in the ‘real-world’. This is when an initiative exits from the original researcher(s) owning the entirety of the impact, to one where the researcher(s) have an ongoing contribution to vastly scaled-up sets of collective impacts that are no longer controlled by any one actor, community or network. Penfield et al.^9^ relates this to ‘knowledge creep’ where new data, information or frameworks become accepted and get absorbed over time.

Careful consideration of how an initiative is developed, emerges, is used, and the resulting benefits is needed to map impact. This process, in its own regard, provides solid foundations for future planning and consideration of possible (or maybe unforeseen) opportunities to develop the impact further as part of ex ante impact forecasting^1,44^. It’s value also lies in communicating and teaching others, using worked case studies, about what impact can mean, to demonstrate how it can evolve and mature, and outline the possible pathways of impact as part of ex post impact assessment^1,44^.

With greater emphasis being placed on impact in research policy and reporting in many parts of the world, it is timely to consider the level of ongoing support required to genuinely capture and assess impact over yearly and decadal timeframes^20^. Creation of environments and cultures in which impact can be incubated, nourished and supported aids effective planning, knowledge translation and engagement. Ongoing research is required to consider, more broadly and laterally, what is measured, what indicators are used, and the evidence required to assign attribution. This remains a challenge not just for the case study documented here, but for the process of impact assessment more generally^1,9^. Continuous monitoring of impacts (both intended and unintended) is needed. To do this requires support and systems to gather, archive and track data, whether quantitative or qualitative, and adequately build evidence portfolios^20^. A keen eye is needed to identify, document and archive evidence that may seem insignificant at the time, but can lead to a step-change in impact, or a re-appearance elsewhere on the pathway.

Impact reporting extends beyond traditional outreach and service roles in academia^16,19^. Despite the increasing recognition of the importance of impact and its permeation into academic lives, it is yet to be formally built into many academic and professional roles^9^. To date, the rewards are implicit rather than explicit^44^. Support is required if impact planning and reporting for assessment is to become a new practice for academics.

Managing the research impact process is vital, but it is also important to be open to new ideas and avenues for creating impact at different stages of the process. It is important to listen and to be attuned to developments outside of academia, and learn to live with the creative spark of uncertainty as we expect the unexpected!
