# Author Correction: Engaging with research impact assessment for an environmental science case study

**DOI:** 10.1038/s41467-019-13141-1

**Published:** 2019-11-08

**Authors:** Kirstie A. Fryirs, Gary J. Brierley, Thom Dixon

**Affiliations:** 1 Department of Environmental Sciences, Macquarie University, Sydney, NSW 2109 Australia; 2 School of Environment, University of Auckland, Auckland, 1010 New Zealand; 3 Research Services, Macquarie University, Sydney, NSW 2109 Australia

**Keywords:** Environmental impact, Research management

Correction to: *Nature Communications* 10.1038/s41467-019-12020-z, published online 4 October 2019.

The original version of this Article has been corrected to include an extended Competing interests declaration: ‘KF and GB are co-developers of the River Styles Framework. River Styles foundation research has been supported through competitive grant schemes and university grants. Consultancy-based River Styles short courses taught by KF and GB are administered by Macquarie University. River Styles contract research is administered by Macquarie University and University of Auckland. River Styles as a trade mark expires in May 2020. TD declares no conflict of interest.’ This replaced the original Competing interests declaration: ‘K.F. and G.B. are co-developers of the River Styles Framework. T.D. declares no competing interests.’ This has been corrected in both the PDF and HTML versions of the Article.

